# Personality and Psychopathology in Adults with Noonan Syndrome

**DOI:** 10.1007/s10880-019-09659-7

**Published:** 2019-09-27

**Authors:** Renée L. Roelofs, Ellen Wingbermühle, Paul T. van der Heijden, Rosella Jonkers, Marieke de Haan, Roy P. C. Kessels, Jos I. M. Egger

**Affiliations:** 1 Centre of Excellence for Neuropsychiatry, Vincent van Gogh Institute for Psychiatry, P.O. Box 5, 5800 AA Venray, The Netherlands; 2 Donders Institute for Brain, Cognition and Behaviour, Radboud University, Nijmegen, The Netherlands; 3 Reinier van Arkel Mental Health Institute, ‘s-Hertogenbosch, The Netherlands; 4 Dimence Group, Deventer, Zwolle, The Netherlands; 5 Het Rughuis, Nijmegen, The Netherlands; 6 Centre of Excellence for Korsakoff and Alcohol-Related Cognitive Disorders, Vincent van Gogh Institute for Psychiatry, Venray, The Netherlands; 7 Department of Medical Psychology, Radboud University Medical Center, Nijmegen, The Netherlands; 8 Stevig, Specialized and Forensic Care for Patients with Intellectual Disabilities, Dichterbij, Oostrum The Netherlands

**Keywords:** Neurodevelopmental disorders, RASopathies, Contextual neuropsychology, Alexithymia, MMPI-2-RF

## Abstract

This is the first controlled study regarding personality and psychopathology in adults with Noonan syndrome (NS). Anxiety, depression, alexithymia and symptoms of Attention Deficit-Hyperactivity Disorder and Autism Spectrum Disorder, have been previously described in NS. More information regarding personality and psychopathology in NS could improve mental health care for this population. Therefore, scores on the Minnesota Multiphasic Personality Inventory-2-Restructured Form (MMPI-2-RF), a widely used self-report questionnaire of personality and psychopathology, were compared between patients with NS (*n *= 18) and matched, healthy controls (*n *= 18). Furthermore, correlations between MMPI-2-RF scores and alexithymia, measured by the Toronto Alexithymia Scale-20, were investigated. Patients with NS showed significantly higher scores, with medium effect sizes, on MMPI-2-RF scales reflecting infrequent responses (F-r), somatic and cognitive complaints (FBS-r and RBS-r), internalizing problems (EID), demoralization (RCd) and introversion (INTR-r), although the overall profile in both groups was within the non-clinical range. Alexithymia correlated with internalizing problems and negative emotionality in the patient group. In conclusion, patients with NS showed higher levels of introversion, which may predispose them to internalizing problems. These problems were indeed more frequent in patients with NS, especially higher levels of demoralization. Patients may benefit from psychological interventions aimed to decrease internalizing problems, introversion and alexithymia.

## Introduction

Noonan syndrome (NS) is a genetic condition, which is estimated to occur in 1 in 1000 to 2500 live births (Noonan, 2005; Nora, Nora, Sinha, Spangler, & Lubs, 1974). NS is one of the ‘RASopathies’, referring to a group of neurodevelopmental disorders with clinical overlap that are caused by gene mutations resulting in dysregulation of the RAS/mitogen-activated protein kinase (RAS/MAPK) pathway. This signal transduction cascade is involved in several developmental processes in the cell and is crucial for normal development (Allanson & Roberts, 2016; Roberts, Allanson, Tartaglia, & Gelb, 2013; Tidyman & Rauen, 2008). To date, mutations in 17 different genes are associated with NS, of which mutations in *PTPN11* (50%), *SOS1* (10%) and *RAF1* (10%) are most common (Tajan, Paccoud, Branka, Edouard, & Yart, 2018). The phenotype of patients with NS is characterized by distinctive facial features (e.g. ocular hypertelorism, ptosis, low-set posteriorly rotated ears), short stature and congenital heart defects (Allanson & Roberts, 2016). Regarding cognitive functioning, NS is associated with varying degrees of developmental delay and intelligence scores tend to vary from intellectual disability to superior levels of functioning. However, most patients with NS show intellectual abilities within the (low)average range (Pierpont, 2016). In children and adolescents with NS, cognitive difficulties in language functioning, visual processing, motor abilities, attention and executive functioning, memory and social functioning have been found (Pierpont, 2016). Studies regarding cognitive functioning in adults are scarce, but mainly report a lowered speed of information processing, subjective executive difficulties and social cognitive problems (e.g. alexithymia) (Wingbermühle, Egger, Verhoeven, van der Burgt, & Kessels, 2012a; Wingbermühle et al., 2012b).

Patients with NS may more likely develop interpersonal problems or psychopathology than healthy individuals due to the previously mentioned cognitive impairments, as well as psychological risk factors that frequently accompany the syndrome. For instance, most patients with NS report a history of being bullied, which may potentially be associated with having a deviant physical appearance and less social resilience or social competence than peers (Wingbermühle et al., 2012b). Bullying victimization and coping with a chronic physical illness have been related to increased mental health problems, especially anxiety and depressive symptoms (Arseneault, 2018; Green, 2004; Karsdorp, Everaerd, Kindt, & Mulder, 2006; Pinquart & Shen, 2011). Moreover, individuals with a developmental delay are known to be at a higher risk for developing psychopathology, although the issue of ‘diagnostic overshadowing’ (i.e. ascribing all features of an individuals’ presentation to developmental delay) frequently leads to the underdiagnosis of (treatable) mental disorders (Einfeld, Ellis, & Emerson, 2011; Siegel & Smith, 2010).

Regarding psychopathology in NS, internalizing and externalizing behavioural problems have been frequently reported by parents of children with NS (Alfieri et al., 2014; Garg et al., 2017; Pierpont, Tworog-Dube, & Roberts, 2015; Sarimski, 2000; Van der Burgt et al., 1999; Wood, Massarano, Super, & Harrington, 1995). Although anxiety and mood problems have been described rather frequently (Noonan, 2005; Smpokou, Tworog-Dube, Kucherlapati, & Roberts, 2012; Wingbermühle et al., 2012a; Pierpont et al., 2015; Perrino et al., 2018) and the prevalence of symptoms of Attention Deficit-Hyperactivity Disorder and Autism Spectrum Disorder seems to be higher in children and adolescents with NS than in the general population (Adviento et al., 2014; Alfieri et al., 2014; Garg et al., 2017; Perrino et al., 2018; Pierpont et al., 2015; Smpokou et al., 2012), only few systematic studies regarding psychopathology in adults with NS are yet available. In a previous study of our research group, significantly higher levels of self-reported distress in social situations—but equal levels of social avoidant behaviour—were found in adults with NS in comparison with controls, as well as higher levels of anger-hostility and obsessive–compulsive complaints, mostly reflecting subjective cognitive difficulties (Wingbermühle et al., 2012a). Furthermore, there are incidental reports of psychopathology in adult patients with NS (i.e. schizophrenia, bipolar disorder, panic disorder, obsessive compulsive disorder, alcoholism, oppositional disorder and anorexia nervosa) (Arvaniti, Samakouri, Keskeridou, & Veletza, 2014; Krishna, Abrams, Taylor, & Behar, 1977; Mahendran & Aw, 1989; Noonan, 2005; Verhoeven et al., 2004; Verhoeven, Wingbermühle, Egger, Van der Burgt, & Tuinier, 2008).

Only a limited number of studies examined personality characteristics in patients with NS. Observations of confident, happy, talkative children, and a socially desirable attitude in adults (friendly, cooperative, willing to please) have been described at an anecdotal level (Collins & Turner, 1973; Verhoeven et al., 2008). Lee, Portnoy, Hill, Gillberg and Patton (2005) found no differences regarding self-esteem in comparison with the general population. In a recent study of Bizaoui, Gage, Brar, Rauen and Weiss (2018), Big Five personality traits of individuals with a RASopathy were compared to that of sibling controls. The 17 patients with NS in the study of Bizaoui et al. (2018) demonstrated lower levels of extraversion and openness to experience in comparison with controls. However, it should be noted that this study mostly consisted of children, only used parent-based reports and no information regarding the level of intellectual functioning was available. In adults with NS, higher levels of alexithymia, reflecting problems in the identification and verbalization of own emotions have been reported in a more systematic study, using matched controls as a reference (Wingbermühle et al., 2012a). Alexithymia has been described as a dimensional personality trait, reflecting weaknesses in the cognitive processing and regulation of one’s own emotions, which is thought to partly overlap with mentalisation (referring to the ability to be aware of and think about the feelings and mental states of oneself and others) (Taylor & Bagby, 2012). Alexithymia can be a risk factor for various medical and psychiatric conditions, and may lead to interpersonal problems (Taylor & Bagby, 2012; Vanheule, Desmet, Meganck, & Bogaerts, 2007). Higher levels of alexithymia in patients with NS may be understood as an inadequate coping strategy (reflecting a proneness to suppressive emotion regulation strategies instead of reappraisal strategies), in which an inability to express feelings may result in mood and anxiety problems (Wingbermühle et al., 2012a).

Although internalizing problems and alexithymia seem to be more prevalent in NS patients, psychopathology and personality characteristics have not yet been systematically studied in adults with NS. Increased insight into the psychopathological issues and personality traits specific to patients with NS may contribute to the behavioural and psychopathological phenotype of NS and could provide targets for counselling and treatment of mental disorders in this population. Therefore, in the current study the results of the Minnesota Multiphasic Personality Inventory-2-Restructured Form (MMPI-2-RF)—a widely used self-report questionnaire of psychopathology and personality traits (Ben-Porath & Tellegen, 2008a, b)—of adults with NS and controls, matched regarding age, sex and intelligence, have been compared. Because symptoms of anxiety and depression have been most consistently reported in studies of adults with NS, higher levels of internalizing problems were expected in the patients with NS in this study in comparison with the control group. More interpersonal problems were expected to be present in the patient group, given the higher levels of alexithymia in patients with NS. Furthermore, to increase the understanding of the typical alexithymic profile of NS patients, we investigated whether the MMPI-2-RF scores were related to alexithymia.

## Methods

### Participants

Eighteen patients with NS and 18 healthy controls (HC) were included in the study. To maximize the comparability between the groups and to facilitate the interpretation of the findings regarding the specific profile of psychopathological vulnerabilities and personality characteristics of patients with NS, control participants were matched to the patient sample regarding age, sex and intelligence (as measured by Wechsler Adult Intelligence Scales). The (social) cognitive abilities of a portion of the participants in the current study have been described in previous studies (Wingbermühle et al., 2012a, b). Participation was voluntary and written informed consent was obtained from all participants and/or their legal representatives. The study was approved by the local Institutional Review Board of the Vincent van Gogh Institute for Psychiatry, in accordance with the Declaration of Helsinki. Individuals with a genetically confirmed or clinical diagnosis of NS, based on the criteria of Van der Burgt (2007), were referred to the Centre of Excellence for Neuropsychiatry of the Vincent van Gogh Institute for (neuro)psychological assessment. Patients were referred for research purposes or when cognitive or psychological complaints were present. The control group consisted of community-dwelling volunteers. Control participants with a diagnosis of a neurological disorder, language barriers or medical problems that might interfere with (neuro)psychological functioning were excluded.

Due to the careful matching procedure, no significant differences between the groups were present with regard to age, MdnNS = 29.5 (range 17–47), MdnHC = 28 (range 18–49), *U *= 172.50, *z *=0.33, *p *= 0.74, *r *= 0.06), sex, χ^2^(1) = 0.12, *p *= 0.73 and full scale IQ (FSIQ) measured by the third or fourth edition of the Dutch version of the Wechsler Adult Intelligence Scale (Wechsler, 2005, 2012) (MdnNS = 93, MdnHC = 93.5, *U *= 157.00, *z *=− 0.16, *p *= 0.89, *r *= − 0.03). FSIQ ranged from 73 to 124 for patients with NS and from 84 to 109 for control participants. Education level, according to the Dutch educational system and ranging from category 1 (less than 6 years of primary education) to 7 (academic degree) (Duits & Kessels, 2014), varied from 3 to 7 in the patient group (mode = 5) and from 4 to 5 in the control group (mode = 5). For individuals with FSIQ scores below 85 (NS *n *= 4, HC *n *= 2), verbal reasoning abilities and comprehension were judged to be sufficient to complete the MMPI-2(-RF) and the test administrator was available to answer questions concerning the items. All patients and controls at least completed 6 years of primary education (in regular or special educational schools), which reflects sufficient reading abilities for administration of the MMPI-2-RF (Ben-Porath & Tellegen, 2008a, b). Moreover, the MMPI-2-RF profiles of these individuals were valid, based on the scores on the Validity scales. There was no significant difference between the groups regarding the average level of alexithymia as measured by the total score of the Toronto Alexithymia Scale-20 (TAS-20) (Bagby, Parker, & Taylor, 1994a; Bagby, Taylor, & Parker, 1994b) (MdnNS = 48.5, MdnHC = 49.0, *U *= 143.00, *z *= − 0.60, *p *= 0.56, *r *= − 0.10). However, four patients with NS (22.2%) showed total scores on the TAS-20 indicative of (high) alexithymia, whereas only one HC showed scores above the threshold of 61 (5.6%). In the patient group, 33% males were included (*n *= 6), compared to 39% in the control group (*n *=7). In 55.6% of the patients, a mutation in the *PTPN11* gene was found (*n *= 10), 22.2% had a mutation in *SOS1* (*n *= 4), 5.6% in *CBL* (*n *= 1), 5.6% in *A2ML1* (*n *= 1) and in 11.1% the genetic mutation was unknown (*n *= 2). A history of bullying victimization was present in 83.3% of the patients and in 38.9% of controls.

### Measures

The MMPI-2-RF is a well-known self-report instrument for measuring psychological symptoms and maladaptive personality traits. The questionnaire consists of 338 true-or-false items, from which 8 Validity scales, 3 Higher-Order (H-O) scales, 9 RC scales, 23 Specific Problem scales, 2 Interest scales and 5 revised Personality Psychology Five (PSY-5) scales can be calculated (Ben-Porath & Tellegen, 2008a, b). The MMPI-2-RF was used because of its correspondence with contemporary models of personality and psychopathology (Kotov et al., 2017). In the present study, the Validity scales, H-O scales, RC scales, Interpersonal Scales and PSY-5 scales of the Dutch language version of the MMPI-2-RF were used (see Table 1 for a description of the scales) (Van der Heijden, Egger & Derksen, 2008; Van der Heijden, Egger, Rossi, & Derksen, 2012a; Van der Heijden, Egger, Rossi, Grundel, & Derksen, 2012b; Van der Heijden, Egger, Rossi, Van der Veld, & Derksen, 2013). For three patients, these scores were based on the MMPI-2-RF booklet; for the other patients and controls, the MMPI-2-RF scores were derived from the original 567-item MMPI-2 booklet (Tellegen & Ben-Porath, 2008; Van der Heijden, Egger, & Derksen, 2010). In addition to the traditional scales in the Dutch language version of the MMPI-2-RF, the Response Bias Scale (RBS)—a scale sensitive to cognitive response bias—was calculated manually using the scoring method of Gervais, Ben-Porath, Wygant and Green (2007) and Gervais, Ben-Porath, Wygant and Sellbom (2010). Because the RBS is not included in the Dutch language version of the MMPI-2-RF, no normative data were available and *T*-scores could therefore not be calculated.

**Table 1:** MMPI-2-RF Scales used in the current study

	Meaning of the scale
Validity scales	
VRIN-r	Variable Response Inconsistency: Random responding
TRIN-r	True Response Inconsistency: Fixed responding
F-r	Infrequent Responses: Responses infrequent in the general population
Fp-r	Infrequent Psychopathology Responses: Responses infrequent in psychiatric populations
Fs-r	Infrequent Somatic Responses: Somatic complaints infrequent in medical patient populations
FBS-r	Symptom Validity: Non-credible somatic and cognitive complaints
L-r	Uncommon Virtues: Rarely claimed moral attributes or activities
K-r	Adjustment Validity: Uncommonly high level of psychological adjustment
RBS-r	Response Bias Scale: Cognitive response bias, overreporting of cognitive complaints
Higher-Order scales	
EID	Emotional/Internalizing Dysfunction: Problems associated with mood and affect
THD	Thought Dysfunction: Problems associated with disordered thinking
BXD	Behavioural/Externalizing Dysfunction: Problems associated with under-controlled behaviour
Restructured Clinical scales	
RCd	Demoralization: General unhappiness and dissatisfaction
RC1	Somatic Complaints: Diffuse physical health complaints
RC2	Low Positive Emotions: A distinctive, core vulnerability factor in depression
RC3	Cynism: Non-self-referential beliefs that others are bad and not to be trusted
RC4	Antisocial Behaviour: Rule breaking and irresponsible behaviour
RC6	Ideas of Persecution: Self-referential beliefs that others pose a threat
RC7	Dysfunctional Negative Emotions: Maladaptive anxiety, anger and irritability
RC8	Aberrant Experiences: Unusual perceptions or thoughts associated with psychosis
RC9	Hypomanic Activation: Over-activation, aggression, impulsivity and grandiosity
Interpersonal scales	
FML	Family Problems: Conflictual family relationships
IPP	Interpersonal Passivity: Being unassertive and submissive
SAV	Social Avoidance: Avoiding or not enjoying social events
SHY	Shyness: Bashful, prone to feel inhibited and anxious around others
DSF	Disaffiliativeness: Disliking people and being around them
Personality Psychopathology Five scales	
AGGR-r	Aggressiveness: Instrumental, goal-directed aggression
PSYC-r	Psychoticism: Disconnection from reality
DISC-r	Disconstraint: Under-controlled behaviour
NEGE-r	Negative Emotionality/Neuroticism: Anxiety, insecurity, worry and fear
INTR-r	Introversion/Low Positive Emotionality: Social disengagement and anhedonia

From Gervais et al. (2007)

The TAS-20 is the most widely used self-report questionnaire for assessing alexithymia (Bagby et al., 1994a, b). The TAS-20 consists of 20 items, rated on a five-point Likert scale, and it provides a total score and three factor scores: Difficulty Identifying Feelings (the ability to identify feelings and distinguish these from somatic sensations that accompany emotional arousal), Difficulty Describing Feelings (the ability to describe feelings to other people) and Externally Oriented Thinking (a cognitive style that shows a preference for external information rather than inner experiences, such as feelings or fantasies) (Parker, Taylor, & Bagby, 2003; Taylor, Bagby, & Parker, 2003). Total scores of 61 and higher are considered to be indicative of (high) alexithymia (Parker et al., 2003). Numerous studies have supported the reliability and validity of the TAS-20 (Bagby et al., 1994a, b; Parker et al., 2003; Taylor et al., 2003). The internal consistency of the total score of the TAS-20 is considered good (Sekely, Bagby, & Porcelli, 2018; Bagby et al., 1994a), and was acceptable in the current sample (Cronbach’s *α* = 0.74).

### Design and Procedure

MMPI-2-RF profiles of 19 patients with NS were available. The validity of the individual MMPI-2-RF profiles was checked and patients were only included when the Cannot Say raw score was lower than 15 and when (U/L) *T*-scores of VRIN-r and TRIN-r were equal to or lower than 80 (Ben-Porath & Tellegen, 2008a). This led to the exclusion of one patient with NS due to a high VRIN-r score. In addition, the scores on the remaining validity scales were carefully inspected regarding the criteria of Ben-Porath and Tellegen (2008a) (F-r ≤ 120, Fp-r ≤ 100, Fs-r ≤ 100, FBS-r ≤ 100, L-r ≤ 80 and K-r ≤ 70). One patient with NS had a Fs-r score higher than 100, but was not excluded, because this score could reflect the actual somatic complications accompanying NS and all other validity scores of this individual were below the cut-off scores, which implicated a valid profile. Two patients and two controls scored between 70 and 75 on the K-r scale, which could indicate underreporting (Ben-Porath & Tellegen, 2008a), but since the other validity scales did not show deviant scores, these profiles were considered valid and were therefore not excluded from the study. This resulted in the inclusion of 18 patients with NS and 18 matched controls.

In total, there were 18 missing answers in the patient group and five in the control group. The missing items in the patient group included 10 items that were part of the Validity scales (VRIN-r, F-r, FBS-r, Fp-r, Fs-r, RBS-r), three of the H-O scales (EID, BXD), 13 of the RC scales (RCd, RC1–4, RC7, RC9), two of the Interpersonal scales (FML) and four of the PSY-5 scales (AGGR-r, DISC-r, PSYC-r, INTR-r). The missing answers in the control group constituted of six items of the Validity scales (VRIN-r, TRIN-r, F-r), three of the H-O scales (EID, BXD), four of the RC scales (RCd, RC2, RC9), two of the interpersonal scales (SHY, SAV) and two of the PSY-5 scales (INTR-r, DISC-r). Because more items were missing in the patient group and could therefore not be included in the analyses, raw scores may be lower for patients with NS than controls. Raw scale scores were used in the analyses.

The current study has a case–control design, studying differences in MMPI-2-RF scores between patients with NS and HC. First, the raw scores on the nine Validity scales are compared to investigate whether differences in response tendencies are present between the groups. Second, the raw scores on the three H-O scales, nine RC scales, five Interpersonal scales and five PSY-5 scales will be compared across the groups. Furthermore, mean (U/L) *T*-scores—calculated using a Dutch normative sample—of the two groups will be investigated qualitatively with regard to elevations (scores ≥ 65) or decreased scores (< 39) on the H-O, RC, Interpersonal and PSY-5 scales. Finally, the correlation between the total score on the TAS-20 (i.e. alexithymia) and the raw scores on the H-O, RC, Interpersonal and PSY-5 scales will be calculated.

### Data Analysis

Due to the violation of normality and the presence of outliers, non-parametric tests are used. Mann–Whitney *U* tests will be performed to investigate differences in raw scores between the groups on the Validity scales, H-O scales, RC scales, Interpersonal scales and PSY-5 scales. Effect sizes are calculated manually, using the following equation: *r *=* z*/(*√N*) (Perdices, 2018; Rosenthal, 1991). Spearman’s correlation coefficients will be calculated to investigate the relation between alexithymia and the scores on the H-O and PSY-5 scales. All analyses are performed with SPSS Version 23.0.0.0.

## Results

On the Validity Scales, patients with NS showed significantly higher scores on F-r, FBS-r and RBS-r than controls, reflecting a tendency to report more psychological, cognitive and somatic complaints (see Table 2). Significantly higher scores were found on the H-O scale Emotional/Internalizing Dysfunction (EID) and the RC scale Demoralization (RCd) in patients with NS compared with controls (see Table 3). No significant differences were found regarding the Interpersonal scales, although Family Problems (FML) showed a medium effect size. Patients with NS showed significantly higher scores than controls on the PSY-5 scale Introversion/Low Positive Emotionality (INTR) and the effect was medium-sized. The differences on Negative Emotionality/Neuroticism (NEGE) were not significant, but showed a medium effect size (see Table 4).

**Table 2:** Results of the MMPI-2-RF Validity Scales of patients with Noonan syndrome (NS, *n* = 18) and healthy controls (HC, *n* = 18)

MMPI-2-RF Validity Scales	Median raw scores (range)		Mean uniform or linear *T*-scores (SD)		*U*	*z*	*p*	*r*
	NS	HC	NS	HC				
VRIN-r	4 (0–7)	3.5 (0–6)	53.94 (11.55)	50.67 (10.30)	133.00	− .93	.37	− .16
TRIN-r	12 (9–12)	11 (8–13)	55.39 (8.13)	51.00 (10.24)	131.50	− 1.02	.34	− .17
F-r	4.5 (1–16)	2.5 (0–10)	66.56 (18.79)	55.17 (13.34)	96.00	− 2.10	.04	− .35
Fp-r	2 (0–6)	1 (0–3)	54.89 (12.48)	51.11 (8.32)	137.00	− .81	.44	− .14
Fs-r	2 (0–9)	1.5 (0–3)	61.17 (19.75)	52.83 (7.78)	130.50	− 1.02	.32	− .17
FBS-r	14 (4–22)	10 (6–17)	68.67 (15.66)	57.33 (8.50)	84.50	− 2.46	.01	− .41
L-r	4 (1–10)	4 (1–9)	47.94 (9.08)	47.00 (10.31)	147.00	− .48	.65	− .08
K-r	6 (1–14)	7 (1–13)	43.94 (14.20)	47.61 (12.53)	190.5	.91	.37	.15
RBS-r	10* (4–16)	7* (3–12)	–	–	96.00	− 2.10	.04	− .35

*VRIN-r* Variable Response Inconsistency, *TRIN-r* True Response Inconsistency, *F-r* Infrequent Responses, *Fp-r* Infrequent Psychopathology Responses, *Fs-r* Infrequent Somatic Responses, *FBS-r* Symptom Validity, *L-r* Uncommon Virtues, *K-r* Adjustment Validity, *RBS-r* Response Bias Scale, *U* Mann–Whitney’s *U* statistic, *z z*-score, *r* effect size estimate

*No normative data were available for the RBS-r, although both scores were below the cut-off score of 17 indicative for exaggeration of memory complaints (Gervais et al., 2010)

**Table 3:** Results of the MMPI-2-RF Higher-Order and Restructured Clinical Scales of patients with Noonan syndrome (NS, *n* = 18) and healthy controls (HC, *n* = 18)

MMPI-2-RF H-O and RC scales	Median raw score (range)		Mean uniform or linear *T*-scores (SD)		*U*	*z*	*p*	*r*
	NS	HC	NS	HC				
EID	17.5 (2–36)	9 (4–34)	61.56 (13.72)	53.44 (10.77)	99.00	− 2.00	.05	− .33
THD	3 (0–8)	1.5 (0–7)	53.72 (11.47)	52.89 (10.69)	154.50	− .24	.82	− .04
BXD	3.5 (0–15)	3.5 (0–14)	54.17 (17.35)	57.00 (14.97)	184.00	.70	.50	.12
RCd	10 (0–23)	2 (0–20)	63.78 (13.21)	52.44 (11.71)	83.50	− 2.49	.01	− .42
RC1	8.5 (0–21)	6.5 (3–14)	64.72 (18.16)	57.83 (8.51)	123.00	− 1.24	.23	− .21
RC2	8 (2–12)	6 (2–13)	55.50 (9.62)	51.94 (8.76)	120.00	− 1.34	.19	− .22
RC3	4 (0–14)	6.5 (0–11)	47.28 (14.42)	50.11 (9.25)	206.00	1.40	.17	.23
RC4	4 (0–18)	2 (0–10)	61.89 (18.26)	56.11 (14.26)	129.00	− 1.05	.31	− .18
RC6	3 (0–10)	2 (0–6)	58.94 (14.72)	56.17 (9.32)	134.50	− .89	.39	− .15
RC7	7.5 (0–16)	5.5 (1–16)	57.72 (13.94)	55.78 (11.53)	144.00	− .57	.58	− .10
RC8	2 (0–5)	2 (0–9)	52.22 (10.76)	54.33 (11.83)	168.00	.19	.86	.03
RC9	7.5 (0–19)	10.5 (1–20)	49.50 (14.46)	54.67 (12.62)	198.00	1.14	.27	.19

*H-O* Higher-Order, *RC* Restructured Clinical, *EID* Emotional/Internalizing Dysfunction, *THD* Thought Dysfunction, *BXD* Behavioural/Externalizing Dysfunction, *RCd* Demoralization, *RC1* Somatic Complaints, *RC2* Low Positive Emotions, *RC3* Cynism, *RC4* Antisocial Behaviour, *RC6* Ideas of Persecution, *RC7* Dysfunctional Negative Emotions, *RC8* Aberrant Experiences, *RC9* Hypomanic Activation, *U* Mann–Whitney’s *U* statistic, *z z*-score, *r* effect size estimate

**Table 4:** Results of the MMPI-2-RF Interpersonal Scales and Personality Psychopathology Five Scales of patients with Noonan syndrome (NS, *n* = 18) and healthy controls (HC, *n* = 18)

MMPI-2-RF Interpersonal and PSY-5 scales	Median raw score (range)		Mean uniform or linear *T*-scores (SD)		*U*	*z*	*p*	*r*
	NS	HC	NS	HC				
FML	2 (0–9)	0 (0–4)	59.61 (19.25)	47.56 (10.04)	106.00	− 1.88	.08	− .31
IPP	4 (1–9)	4 (0–8)	52.83 (9.81)	52.06 (9.55)	155.50	− .21	.84	− .04
SAV	4 (0–8)	3.5 (0–7)	46.83 (8.08)	45.67 (8.00)	149.50	− .40	.70	− .07
SHY	3 (0–7)	4 (0–7)	54.17 (13.01)	54.06 (9.38)	167.50	.18	.86	.03
DSF	0 (0–3)	0 (0–1)	48.44 (9.20)	44.33 (3.88)	130.00	− 1.47	.32	− .25
AGGR-r	7.5 (0–15)	7.5 (3–14)	49.17 (12.78)	49.39 (9.59)	170.50	.27	.79	.05
PSYC-r	3 (0–8)	3 (0–8)	53.89 (11.88)	54.72 (10.81)	167.50	.18	.86	.03
DISC-r	3.5 (0–14)	4 (0–13)	49.56 (14.13)	53.56 (14.50)	197.50	1.14	.27	.19
NEGE-r	8.5 (1–17)	5.5 (2–13)	62.28 (14.64)	54.06 (9.50)	105.50	− 1.79	.07	− .30
INTR-r	9 (1–14)	7 (2–14)	51.67 (8.37)	45.67 (8.88)	96.50	− 2.08	.04	− .35

*FML* Family Problems, *IPP* Interpersonal Passivity, *SAV* Social Avoidance, *SHY* Shyness, *DSF* Disaffiliativeness, *AGGR-r* Aggressiveness, *PSYC-r* Psychoticism, *DISC-r* Disconstraint, *NEGE-r* Negative Emotionality/Neuroticism, *INTR-r* Introversion/Low Positive Emotionality, *U* Mann–Whitney’s *U* statistic, *z z*-score, *r* effect size estimate

**Fig. 1:**
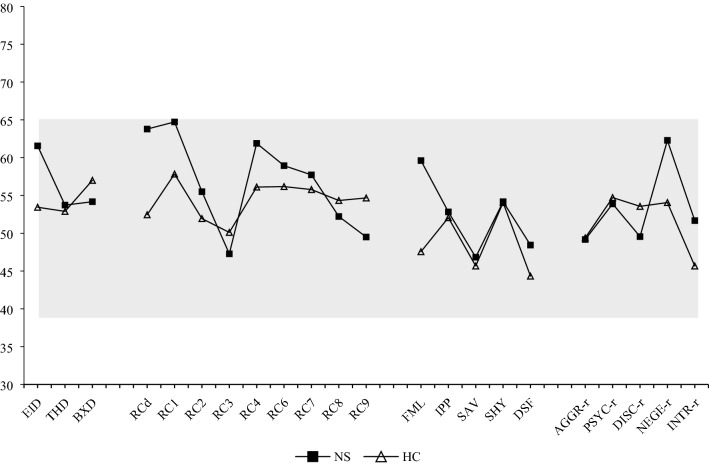
Mean MMPI-2-RF (U/L) *T*-scores of patients with NS and healthy controls. *Note*. *EID* Emotional/Internalizing Dysfunction, *THD* Thought Dysfunction, *BXD* Behavioural/Externalizing Dysfunction, *RCd* Demoralization, *RC1* Somatic Complaints, *RC2* Low Positive Emotions, *RC3* Cynism, *RC4* Antisocial Behaviour, *RC6* Ideas of Persecution, *RC7* Dysfunctional Negative Emotions, *RC8* Aberrant Experiences, *RC9* Hypomanic Activation, *FML* Family Problems, *IPP* Interpersonal Passivity, *SAV* Social Avoidance, *SHY* Shyness, *DSF* Disaffiliativeness, *AGGR-r* Aggressiveness, *PSYC-r* Psychoticism, *DISC-r* Disconstraint, *NEGE-r* Negative Emotionality/Neuroticism, *INTR-r* Introversion/Low Positive Emotionality

The presentation of the mean (U/L) *T*-scores in Fig. 1 and Tables 2, 3 and 4 shows that the scores in both groups are within the normal, non-clinical, range. In general, the scores in the control group seem to be lower than in the patient group. While Emotional/Internalizing Dysfunction shows the highest *T*-score of the H-O scales in the patient group, Behavioural/Externalizing Dysfunction is highest in the control group. Regarding the RC scales, it appears that *T*-scores for Somatic Complaints and Demoralization almost reach the (clinical) threshold of 65 in the patient group, and that the score for Antisocial Behaviour is the next highest score within the profile. Inspection of the item scores of the scale Antisocial Behaviour revealed that patients especially reported family problems more frequently than controls. Cynism and Hypomanic Activation show the lowest *T*-scores in the patient group. In the control group the highest *T*-scores are found for Somatic Complaints, Ideas of Persecution and Antisocial Behaviour. The lowest score is for Cynism. In patients with NS, Family Problems shows the highest *T*-score within the Interpersonal scales while Social Avoidance demonstrates the lowest score. In the control group, the highest *T*-score is found for Shyness, and the lowest for Disaffiliativeness. Regarding the PSY-5 scales, patients with NS show the highest *T*-score for Negative Emotionality/Neuroticism, and the lowest for Aggressiveness. For the control participants, Psychoticism shows the highest *T*-score, while Introversion/Low Positive Emotionality demonstrates the lowest score.

After calculating Spearman’s correlation coefficients between the total score on the TAS-20 and the H-O and PSY-5 scales of the MMPI-2-RF for patients with NS and controls separately, significantly large positive relations between alexithymia and EID and Negative Emotionality/Neuroticism were found in the patient group. The medium-sized correlations between the TAS-20 and THD and AGGR-r were statistically not significant. In the control group, the medium-sized correlations between the TAS-20 and EID, BXD and PSYC-r were statistically not significant (see Table 5).

**Table 5:** Spearman’s correlation coefficients between the total score of the TAS-20 and raw scores on the MMPI-2-RF Higher-Order Scales and PSY-5 Scales for patients with Noonan syndrome (NS) and Healthy controls (HC)

MMPI-2-RF scales	NS (*n* = 18)		HC (*n* = 18)	
	*r*	*p*	*r*	*p*
EID	.52	.03	.43	.07
THD	.30	.23	.11	.65
BXD	.27	.28	.35	.16
AGGR-r	.30	.23	− .10	.70
PSYC-r	.16	.52	.30	.22
DISC-r	.00	> .99	.27	.29
NEGE-r	.50	.03	.23	.37
INTR-r	.19	.45	.13	.60

*EID* Emotional/Internalizing Dysfunction, *THD* Thought Dysfunction, *BXD* Behavioural/Externalizing Dysfunction, *AGGR-r* Aggressiveness, *PSYC-r* Psychoticism, *DISC-r* Disconstraint, *NEGE-r* Negative Emotionality/Neuroticism, *INTR-r* Introversion/Low Positive Emotionality

## Discussion

This is the first controlled study investigating personality characteristics and psychopathology in adult patients with NS, using the MMPI-2-RF, a well-known and widely used comprehensive instrument for this purpose. Patients with NS showed higher scores on three validity scales, reflecting a higher amount of psychological, cognitive and somatic complaints. The H-O scales showed higher levels of internalizing problems in patients with NS. Regarding the RC scales, only a higher score on Demoralization was found in the patient group. There were no significant differences regarding the Interpersonal scales, while on the PSY-5 scales patients with NS showed significantly higher scores on introversion than controls. Alexithymia was significantly correlated with negative emotionality and internalizing problems in patients with NS.

Our finding of more subjectively reported psychological, cognitive and somatic problems in patients with NS, as reflected by the Validity scales of the MMPI-2-RF, can be understood given the fact that NS is a complex system disorder, affecting somatic, cognitive and psychological functioning. Higher scores on these scales may also suggest a tendency to over-report symptoms, although other Validity Scales (Fp-r, Fs-r) reflecting complaints that are rarely endorsed by individuals with severe psychopathology or medical complaints were not higher in patients with NS than in controls. This suggests that the elevated scores in this study reflect genuinely experienced difficulties in NS. Another explanation for finding higher levels of reported somatic problems in patients with NS may be related to alexithymia, as difficulties in identifying and verbalizing feelings have been associated repeatedly with somatization (Mattila et al., 2008). However, there were no differences in the mean level of alexithymia between the two groups in this study.

The higher levels of emotional and internalizing dysfunction in patients with NS are consistent with previous reports of anxiety and mood problems in NS and in accordance with our hypotheses (Noonan, 2005; Perrino et al., 2018; Smpokou et al., 2012; Wingbermühle et al., 2012a). The results of the current study showed that especially higher levels of demoralization were characteristic for the difference between patients with NS and controls. According to Ben-Porath and Tellegen (2008a), demoralization reflects a pervasive and affect-laden dimension of unhappiness and life dissatisfaction. The higher levels of demoralization in NS could reflect the experienced burden of living with this chronic condition, while no specific psychopathology is present, as reflected by the lack of considerable effect sizes on any of the other RC scales. This finding is in contrast with previous reports of an unimpaired quality of life in patients with NS (Binder et al., 2012; Shaw, Kalidas, Crosby, Jeffery, & Patton, 2007). Finding lower levels of life satisfaction in the current study may be explained by the higher face validity of the items of quality of life questionnaires compared to the items of the MMPI-2-RF, which could have triggered a social desirable response tendency in previous studies. Also, the inclusion of patients that were referred for neuropsychological assessment due to psychological or cognitive complaints could explain the higher levels of demoralization in this study. In addition, the percentage of patients reporting bullying victimization was higher than that of controls, which may be related to the development of higher levels of internalizing problems in adulthood in the patients. Studies in typically developing children and adolescents have demonstrated that childhood bullying victimization is associated with withdrawal from social activities and internalizing symptoms (Cillessen & Lansu, 2015; Reijntjes, Kamphuis, Prinzie, & Telch, 2010). Other studies also found that childhood bullying was related to adult mental health problems, among which internalizing problems (Takizawa, Maughan, & Arseneault, 2014; Arseneault, 2018). Last, inspection of the standardized *T*-scores showed results within the normal range in both groups and hence did not reflect clinical levels of psychopathology. In general, the scores of patients seemed to be higher than controls.

No significant differences were found regarding the Interpersonal scales, although the effect size for the difference regarding family problems was medium. With regard to the PSY-5 scales, patients with NS reported higher levels of introversion, reflecting a lack of positive emotional experiences and avoidance of social situations and interactions (Ben-Porath & Tellegen, 2008a). The scores on Negative Emotionality/Neuroticism showed medium effect sizes, but were statistically not significant. Bizaoui et al. (2018) also found lower levels of extraversion in their sample of (mostly) children and adolescents with NS. However, in a previous study of our research group, higher levels of social distress were found in patients with NS, but no social avoidance (Wingbermühle et al., 2012a). A qualitative investigation of the item scores of the Introversion scale in the current study showed that patients with NS especially answered the items regarding the experience of positive emotions more negatively than controls, and not the items reflecting social interactions. Although this could reflect an alexithymic characteristic, the correlation between alexithymia and introversion was only small, and the difference between the groups showed a small (and non-significant) effect. An introverted personality style has been associated with social introversion, anhedonia, restricted interests and a pessimistic outlook (Ben-Porath & Tellegen, 2008a). Hence, introversion is known to be related to mood and (social) anxiety problems (Kotov, Gamez, Schmidt, & Watson, 2010). Possibly, an introverted personality style may predispose patients with NS to internalizing problems.

Because higher levels of alexithymia in adults with NS have been found in previous studies (Wingbermühle et al., 2012a) and alexithymia has been related to psychopathology (Taylor & Bagby, 2012), the correlation between MMPI-2-RF scales and alexithymia, as measured by the TAS-20, was investigated. Higher levels of alexithymia appeared to be related to higher levels of internalizing problems and negative emotionality/neuroticism in patients with NS. The medium-sized positive correlations between the TAS-20 and THD and AGGR-r were not statistically significant. In the control group, the medium-sized positive correlations between the TAS-20 and EID, BXD and PSYC-r were not statistically significant. Finding a positive relation between internalizing problems and alexithymia was expected. Bagby, Taylor and Parker (1994b) found alexithymia to correlate positively with neuroticism and negatively with extraversion in a student sample, suggesting a tendency of alexithymic individuals to be anhedonic and to experience emotions with less differentiation, due to weaknesses in the cognitive representation and modulation of emotions.

Potential limitations of this study should be considered. Because only patients were included who could complete the MMPI-2-RF, the sample consisted of relatively more patients with higher IQ scores—hence more patients with a mutation in *SOS1*. Other studies also found patients with a mutation in *SOS1* to have more favourable outcomes regarding intellectual functioning than patients with *PTPN11* (Pierpont, 2016). Furthermore, patients with psychological or cognitive problems may be overrepresented in this study, because they could be more inclined to participate or were more likely to be referred for neuropsychological assessment, which might have consequences for the generalization of the findings of this study to the general NS population. Also, there were more missing items in the patient group than in the control group, which could have led to an underestimation of problems in the patient group. Moreover, although the TAS-20 is a valid and frequently used instrument for measuring alexithymia, the use of a self-report measure to assess alexithymia could be viewed as paradoxical, because alexithymic individuals may be less able to reflect on their emotional difficulties due to a limited affective insight (Lane, Ahern, Schwartz, & Kaszniak, 1997; Waller & Scheidt, 2004). This could have led to an underestimation of alexithymic problems in the patient group.

The results of this study nonetheless point out the fact that patients with NS show a more introvert personality style and experience more internalizing difficulties in comparison with controls. Moreover, higher levels of alexithymia appeared to be related to higher levels of internalizing problems and negative emotionality in the patient group. Clinicians (general practitioners, clinical (neuro)psychologists, medical specialists) should be aware of the psychological vulnerability of patients with NS, especially when patients have an alexithymic tendency. If these problems interfere with daily functioning, patients with NS can benefit from psychological interventions, aimed to decrease internalizing problems, introversion and alexithymia.

## References

[CR1] Adviento, B., Corbin, I. L., Widjaja, F., Desachy, G., Enrique, N., Rosser, T., … Weiss, L. A. (2014). Autism traits in the RASopathies. *Journal of Medical Genetics, 51*, 10–20. 10.1136/jmedgenet-2013-101951. 10.1136/jmedgenet-2013-101951 PMC4230531 24101678

[CR2] Alfieri, P., Piccini, G., Caciolo, C., Perrino, F., Gambardella, M. L., Mallardi, M., … Vicari, S. (2014). Behavioral profile in RASopathies. *American Journal of Medical Genetics Part A, 164*, 934–942. 10.1002/ajmg.a.36374. 10.1002/ajmg.a.36374 24458522

[CR3] Allanson, J. E., & Roberts, A. E. (2016). Noonan syndrome. In M. P. Adam, H. H. Ardinger, R. A. Pagon, S. E. Wallace, L. J. Bean, K. Stephens & A. Amemiya (Eds.), *GeneReviews*^*®*^. Seattle, WA: University of Washington, Seattle. Retrieved June 1, 2018 from http://www.ncbi.nlm.nih.gov/books/NBK1124/.

[CR4] Arseneault L (2018). Annual Research Review: The persistent and pervasive impact of being bullied in childhood and adolescence: Implications for policy and practice. Journal of Child Psychology and Psychiatry.

[CR5] Arvaniti A, Samakouri M, Keskeridou F, Veletza S (2014). Concurrence of anorexia nervosa and Noonan syndrome. European Eating Disorders Review: The Journal of the Eating Disorders Association.

[CR6] Bagby RM, Parker JD, Taylor GJ (1994). The twenty-item Toronto Alexithymia Scale-I. Item selection and cross-validation of the factor structure. Journal of Psychosomatic Research.

[CR7] Bagby RM, Taylor GJ, Parker JD (1994). The Twenty-item Toronto Alexithymia Scale—II. Convergent, discriminant, and concurrent validity. Journal of Psychosomatic Research.

[CR8] Ben-Porath YS, Tellegen A (2008). MMPI-2-RF manual for administration, scoring, and interpretation.

[CR9] Ben-Porath YS, Tellegen A (2008). MMPI-2-RF user’s guide for reports.

[CR10] Binder, G., Grathwol, S., von Loeper, K., Blumenstock, G., Kaulitz, R., Freiberg, C., … Paul, T. (2012). Health and quality of life in adults with Noonan syndrome. *The Journal of Pediatrics, 161*, 501–505.e1. 10.1016/j.jpeds.2012.02.043. 10.1016/j.jpeds.2012.02.043 22494877

[CR11] Bizaoui V, Gage J, Brar R, Rauen KA, Weiss LA (2018). RASopathies are associated with a distinct personality profile. American Journal of Medical Genetics. Part B, Neuropsychiatric Genetics: The Official Publication of the International Society of Psychiatric Genetics.

[CR12] Cillessen AHN, Lansu TAM (2015). Stability, correlates, and time-covarying associations of peer victimization from grade 4 to 12. Journal of Clinical Child and Adolescent Psychology.

[CR13] Collins E, Turner G (1973). The Noonan syndrome—A review of the clinical and genetic features of 27 cases. The Journal of Pediatrics.

[CR14] Duits, A., & Kessels, R. (2014). Schatten van het premorbide functioneren [Estimating premorbid functioning]. In M. Hendriks, R. Kessels, M. Gorissen, B. Schmand & A. Duits (Eds.), *Neuropsychologische diagnostiek: De klinische praktijk [Neuropsychological assessment: Clinical practice]* (pp. 173–186). Amsterdam: Boom.

[CR15] Einfeld SL, Ellis LA, Emerson E (2011). Comorbidity of intellectual disability and mental disorder in children and adolescents: A systematic review. Journal of Intellectual and Developmental Disability.

[CR16] Garg, S., Brooks, A., Burns, A., Burkitt-Wright, E., Kerr, B., Huson, S., … Green, J. (2017). Autism spectrum disorder and other neurobehavioural comorbidities in rare disorders of the Ras/MAPK pathway. *Developmental Medicine and Child Neurology, 59*, 544–549. 10.1111/dmcn.13394. 10.1111/dmcn.13394 28160302

[CR17] Gervais RO, Ben-Porath YS, Wygant DB, Green P (2007). Development and validation of a response bias scale (RBS) for the MMPI-2. Assessment.

[CR18] Gervais RO, Ben-Porath YS, Wygant DB, Sellbom M (2010). Incremental validity of the MMPI-2-RF over-reporting scales and RBS in assessing the veracity of memory complaints. Archives of Clinical Neuropsychology.

[CR19] Green A (2004). Outcomes of congenital heart disease: A review. Pediatric Nursing.

[CR20] Karsdorp PA, Everaerd W, Kindt M, Mulder BJM (2006). Psychological and cognitive functioning in children and adolescents with congenital heart disease: A meta-analysis. Journal of Pediatric Psychology.

[CR21] Kotov R, Gamez W, Schmidt F, Watson D (2010). Linking “big” personality traits to anxiety, depressive, and substance use disorders: A meta-analysis. Psychological Bulletin.

[CR22] Kotov, R., Krueger, R. F., Watson, D., Achenbach, T. M., Althoff, R. R., Bagby, R. M., … Zimmerman, M. (2017). The Hierarchical Taxonomy of Psychopathology (HiTOP): A dimensional alternative to traditional nosologies. *Journal of Abnormal Psychology, 126*, 454–477. 10.1037/abn0000258. 10.1037/abn0000258 28333488

[CR23] Krishna NR, Abrams R, Taylor MA, Behar D (1977). Schizophrenia in a 46, XY male with the Noonan syndrome. The British Journal of Psychiatry: The Journal of Mental Science.

[CR24] Lane RD, Ahern GL, Schwartz GE, Kaszniak AW (1997). Is alexithymia the emotional equivalent of blindsight?. Biological Psychiatry.

[CR25] Lee DA, Portnoy S, Hill P, Gillberg C, Patton MA (2005). Psychological profile of children with Noonan syndrome. Developmental Medicine and Child Neurology.

[CR26] Mahendran R, Aw SC (1989). Noonan’s syndrome with mental retardation presenting with an affective disorder—Case report. Singapore Medical Journal.

[CR27] Mattila AK, Kronholm E, Jula A, Salminen JK, Koivisto A-M, Mielonen R-L, Joukamaa M (2008). Alexithymia and somatization in general population. Psychosomatic Medicine.

[CR28] Noonan JA, Goldstein S, Reynolds CR (2005). Noonan syndrome. Handbook of neurodevelopmental and genetic disorders in adults.

[CR29] Nora JJ, Nora AH, Sinha AK, Spangler RD, Lubs HA (1974). The Ullrich–Noonan syndrome (Turner phenotype). American Journal of Diseases of Children (1960).

[CR30] Parker JDA, Taylor GJ, Bagby RM (2003). The 20-Item Toronto Alexithymia Scale. III. Reliability and factorial validity in a community population. Journal of Psychosomatic Research.

[CR31] Perdices M (2018). Null hypothesis significance testing, p-values, effects sizes and confidence intervals. Brain Impairment.

[CR32] Perrino, F., Licchelli, S., Serra, G., Piccini, G., Caciolo, C., Pasqualetti, P., …, Vicari, S. (2018). Psychopathological features in Noonan syndrome. *European Journal of Paediatric Neurology: Official Journal of the European Paediatric Neurology Society, 22*, 170–177. 10.1016/j.ejpn.2017.09.009. 10.1016/j.ejpn.2017.09.009 29037749

[CR33] Pierpont EI (2016). Neuropsychological functioning in individuals with Noonan syndrome: A systematic literature review with educational and treatment recommendations. Journal of Pediatric Neuropsychology.

[CR34] Pierpont EI, Tworog-Dube E, Roberts AE (2015). Attention skills and executive functioning in children with Noonan syndrome and their unaffected siblings. Developmental Medicine and Child Neurology.

[CR35] Pinquart M, Shen Y (2011). Behavior problems in children and adolescents with chronic physical illness: A meta-analysis. Journal of Pediatric Psychology.

[CR36] Reijntjes A, Kamphuis JH, Prinzie P, Telch MJ (2010). Peer victimization and internalizing problems in children: A meta-analysis of longitudinal studies. Child Abuse and Neglect.

[CR37] Roberts AE, Allanson JE, Tartaglia M, Gelb BD (2013). Noonan syndrome. Lancet (London, England).

[CR38] Rosenthal R (1991). Meta-analytic procedures for social research.

[CR39] Sarimski K (2000). Developmental and behavioural phenotype in Noonan syndrome?. Genetic Counseling (Geneva, Switzerland).

[CR40] Sekely A, Bagby RM, Porcelli P, Luminet O, Bagby RM, Taylor GJ (2018). Assessment of the alexithymia construct. Alexithymia, advances in research, theory, and clinical practice.

[CR42] Shaw AC, Kalidas K, Crosby AH, Jeffery S, Patton MA (2007). The natural history of Noonan syndrome: A long-term follow-up study. Archives of Disease in Childhood.

[CR43] Siegel MS, Smith WE (2010). Psychiatric features in children with genetic syndromes: Toward functional phenotypes. Child and Adolescent Psychiatric Clinics of North America.

[CR44] Smpokou P, Tworog-Dube E, Kucherlapati RS, Roberts AE (2012). Medical complications, clinical findings, and educational outcomes in adults with Noonan syndrome. American Journal of Medical Genetics. Part A.

[CR45] Tajan M, Paccoud R, Branka S, Edouard T, Yart A (2018). The RASopathy family: Consequences of germline activation of the RAS/MAPK pathway. Endocrine Reviews.

[CR46] Takizawa R, Maughan B, Arseneault L (2014). Adult health outcomes of childhood bullying victimization: Evidence from a five-decade longitudinal British birth cohort. American Journal of Psychiatry.

[CR47] Taylor, G. J., & Bagby, M. (2012). The alexithymia personality dimension. In T. A. Widiger (Ed.), *The Oxford handbook of personality disorders* (1st ed., pp. 1–79). Oxford University Press. 10.1093/oxfordhb/9780199735013.001.0001.

[CR48] Taylor GJ, Bagby RM, Parker JDA (2003). The 20-Item Toronto Alexithymia Scale. IV. Reliability and factorial validity in different languages and cultures. Journal of Psychosomatic Research.

[CR49] Tellegen A, Ben-Porath YS (2008). MMPI-2-RF technical manual.

[CR50] Tidyman WE, Rauen KA (2008). Noonan, Costello and cardio–facio-cutaneous syndromes: Dysregulation of the Ras-MAPK pathway. Expert Reviews in Molecular Medicine.

[CR51] Van der Burgt I (2007). Noonan syndrome. Orphanet Journal of Rare Diseases.

[CR52] Van der Burgt I, Thoonen G, Roosenboom N, Assman-Hulsmans C, Gabreels F, Otten B, Brunner HG (1999). Patterns of cognitive functioning in school-aged children with Noonan syndrome associated with variability in phenotypic expression. The Journal of Pediatrics.

[CR500] Van der Heijden PT, Egger JIM, Derksen JJL (2008). Psychometric evaluation of the MMPI-2 restructured clinical scales in two Dutch samples. Journal of Personality Assessment.

[CR53] Van der Heijden PT, Egger JIM, Derksen JJL (2010). Comparability of scores on the MMPI-2-RF scales generated with the MMPI-2 and MMPI-2-RF booklets. Journal of Personality Assessment.

[CR54] Van der Heijden PT, Egger JIM, Rossi G, Derksen JJL (2012). Integrating psychopathology and personality disorders conceptualized by the MMPI-2-RF and the MCMI-III: A structural validity study. Journal of Personality Assessment.

[CR55] Van der Heijden PT, Egger JIM, Rossi G, Grundel GMP, Derksen JJL (2012). The MMPI-2-Restructured Form and the standard MMPI-2 clinical scales in relation to DSM-IV. European Journal of Psychological Assessment.

[CR56] Van der Heijden PT, Egger JIM, Rossi GMP, Van der Veld WM, Derksen JJL (2013). Personality and psychopathology: Mapping the MMPI-2-RF on Cloninger’s psychobiological model of personality. Assessment.

[CR57] Vanheule S, Desmet M, Meganck R, Bogaerts S (2007). Alexithymia and interpersonal problems. Journal of Clinical Psychology.

[CR58] Verhoeven WM, Hendrikx JL, Doorakkers MC, Egger JI, Van der Burgt I, Tuinier S (2004). Alexithymia in Noonan syndrome. Genetic Counseling.

[CR59] Verhoeven W, Wingbermühle E, Egger J, Van der Burgt I, Tuinier S (2008). Noonan syndrome: Psychological and psychiatric aspects. American Journal of Medical Genetics. Part A.

[CR60] Waller E, Scheidt CE (2004). Somatoform disorders as disorders of affect regulation. Journal of Psychosomatic Research.

[CR61] Wechsler D (2005). WAIS-III technical manual.

[CR62] Wechsler D (2012). Wechsler Adult Intelligence Scale Fourth Edition, Nederlandstalige bewerking (WAIS-IV-NL): Technische handleiding [WAIS-IV Dutch version: Technical manual].

[CR63] Wingbermuhle E, Egger JIM, Verhoeven WMA, van der Burgt I, Kessels RPC (2012). Affective functioning and social cognition in Noonan syndrome. Psychological Medicine.

[CR64] Wingbermühle E, Roelofs RL, van der Burgt I, Souren PM, Verhoeven WMA, Kessels RPC, Egger JIM (2012). Cognitive functioning of adults with Noonan syndrome: A case–control study. Genes, Brain, and Behavior.

[CR65] Wood A, Massarano A, Super M, Harrington R (1995). Behavioural aspects and psychiatric findings in Noonan’s syndrome. Archives of Disease in Childhood.

